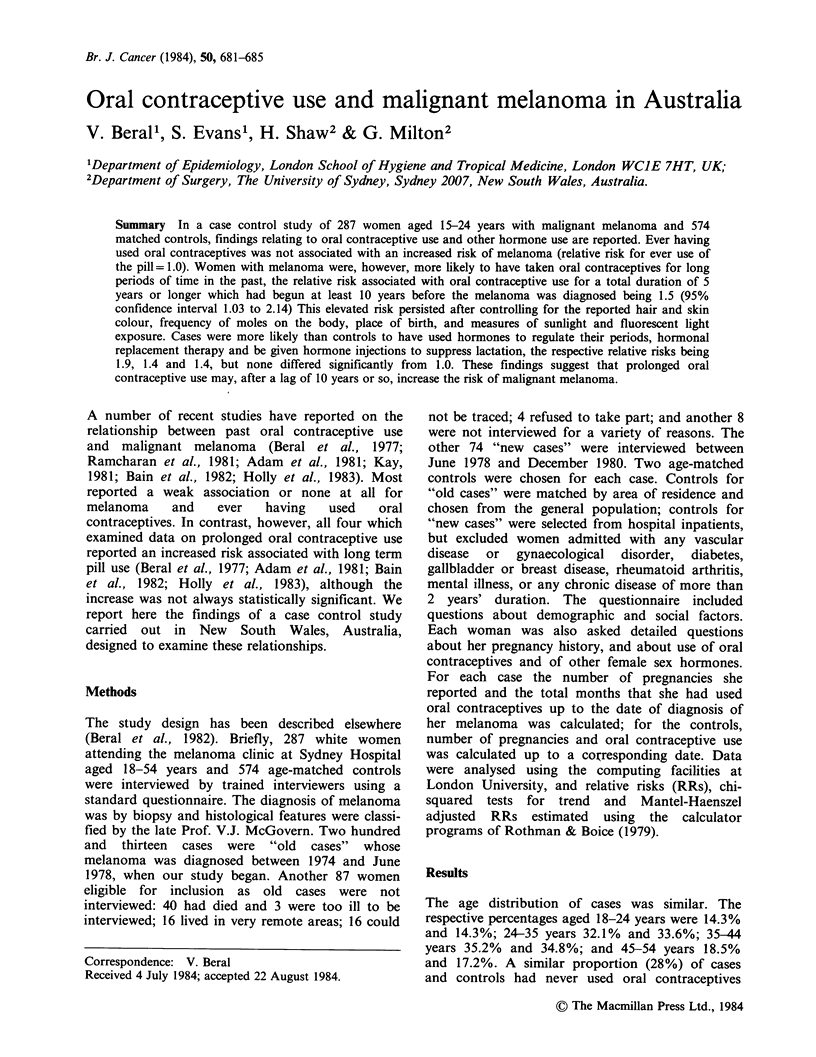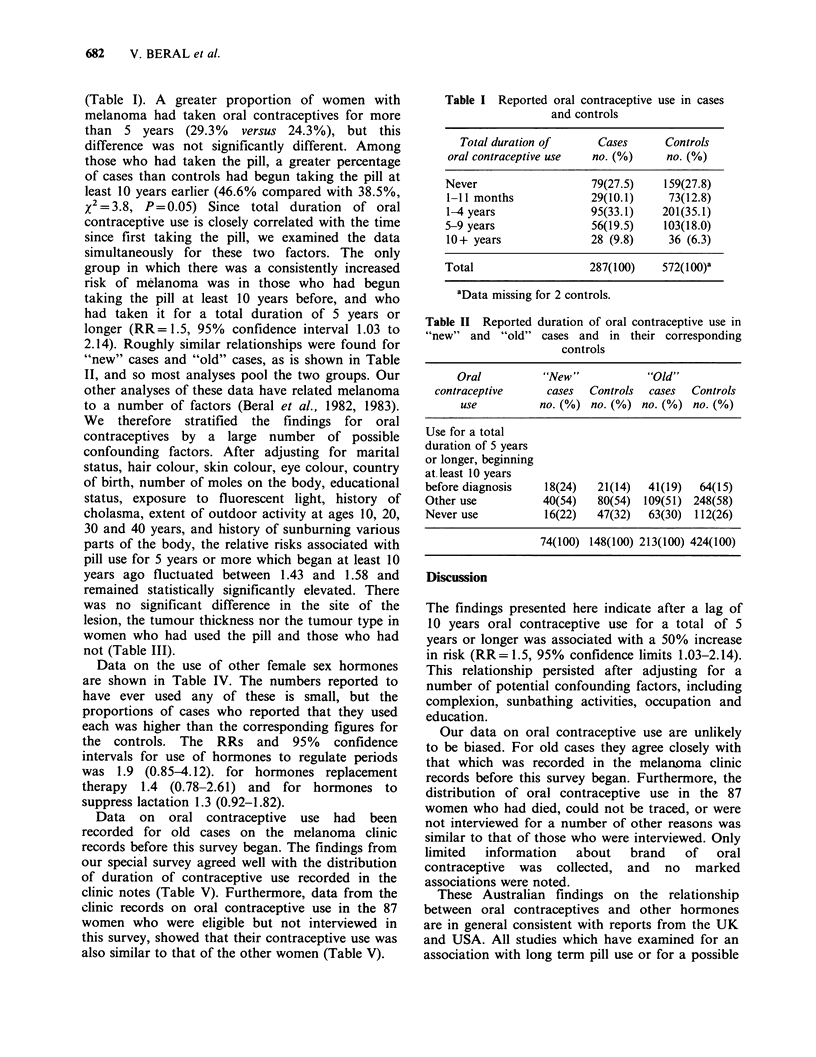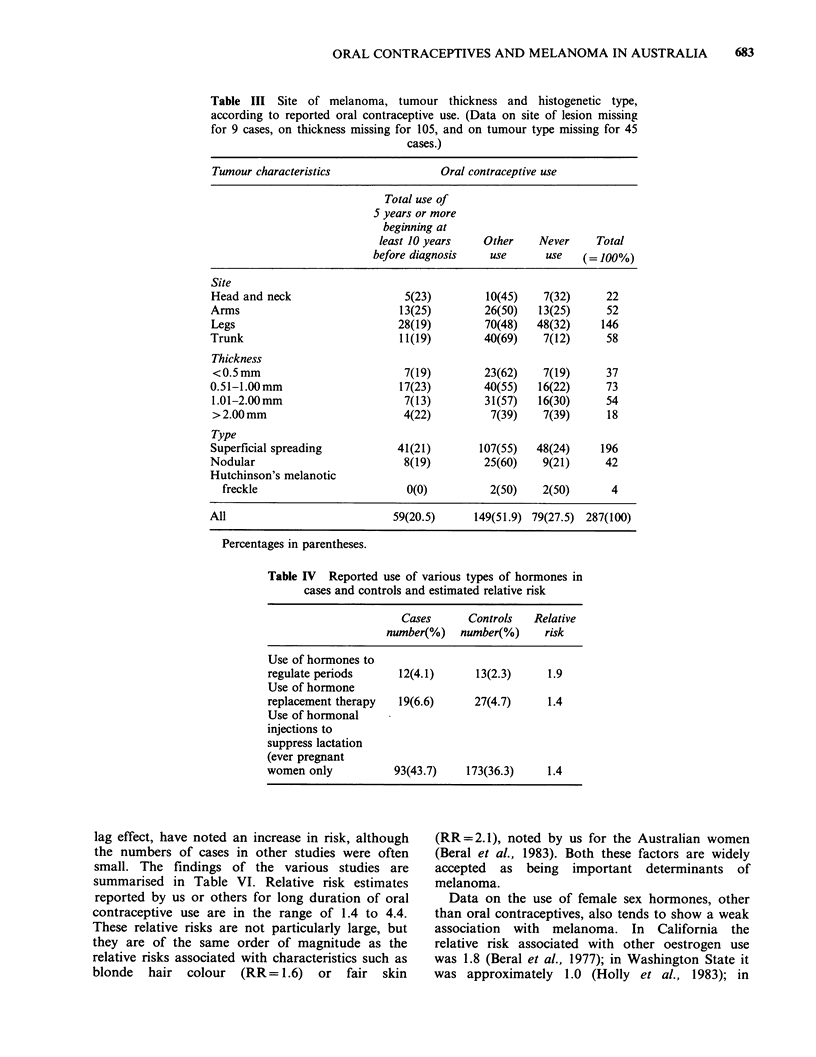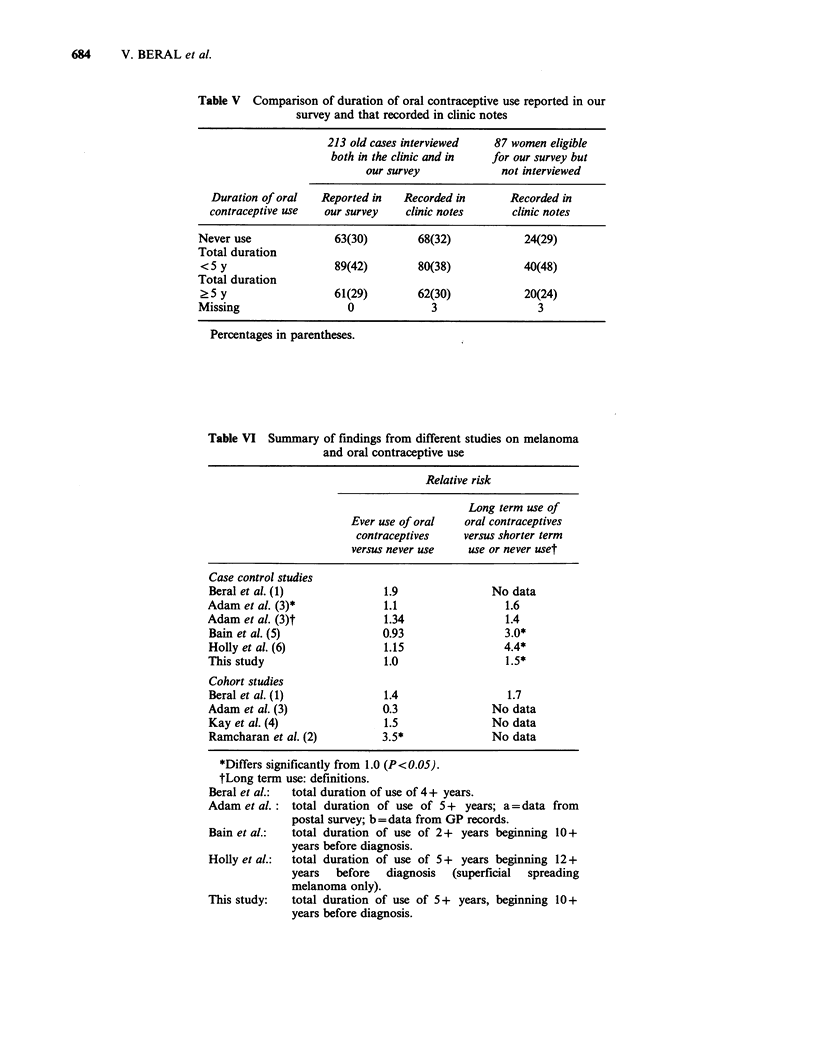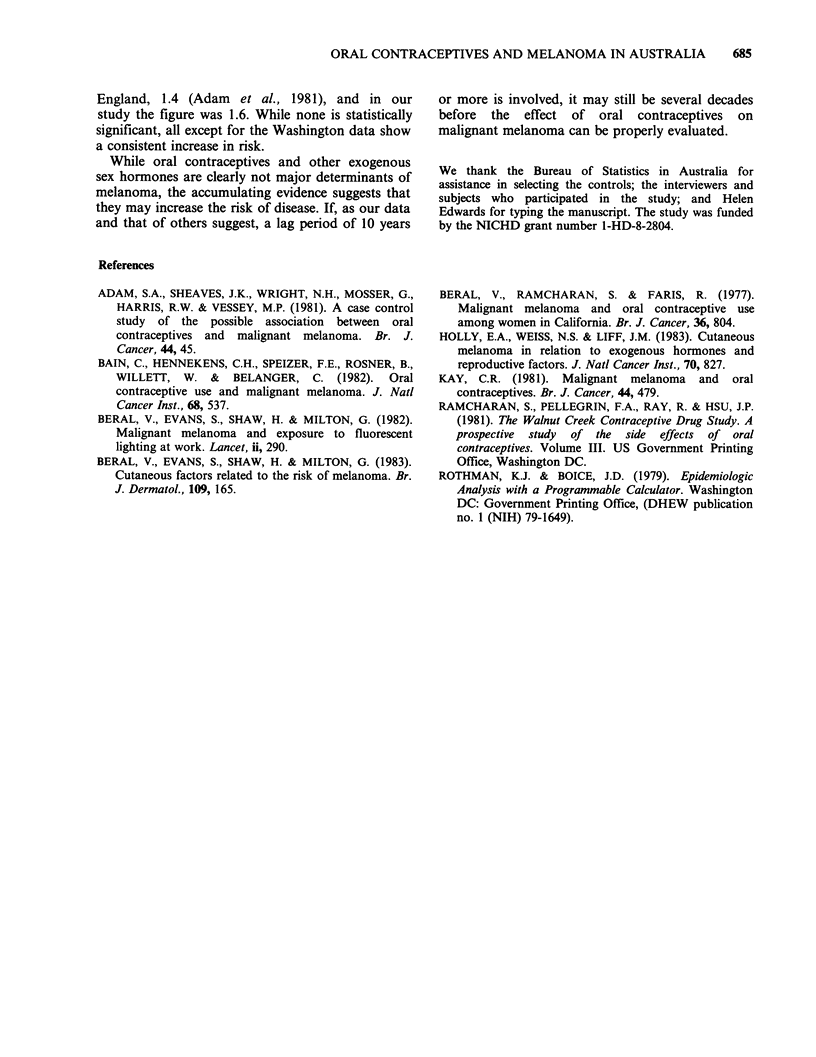# Oral contraceptive use and malignant melanoma in Australia.

**DOI:** 10.1038/bjc.1984.236

**Published:** 1984-11

**Authors:** V. Beral, S. Evans, H. Shaw, G. Milton

## Abstract

In a case control study of 287 women aged 15-24 years with malignant melanoma and 574 matched controls, findings relating to oral contraceptive use and other hormone use are reported. Ever having used oral contraceptives was not associated with an increased risk of melanoma (relative risk for ever use of the pill = 1.0). Women with melanoma were, however, more likely to have taken oral contraceptives for long periods of time in the past, the relative risk associated with oral contraceptive use for a total duration of 5 years or longer which had begun at least 10 years before the melanoma was diagnosed being 1.5 (95% confidence interval 1.03 to 2.14) This elevated risk persisted after controlling for the reported hair and skin colour, frequency of moles on the body, place of birth, and measures of sunlight and fluorescent light exposure. Cases were more likely than controls to have used hormones to regulate their periods, hormonal replacement therapy and be given hormone injections to suppress lactation, the respective relative risks being 1.9, 1.4 and 1.4, but none differed significantly from 1.0. These findings suggest that prolonged oral contraceptive use may, after a lag of 10 years or so, increase the risk of malignant melanoma.


*Br. J. Cancer* (1984), **50**, 681–685

### Oral contraceptive use and malignant melanoma in Australia

V. Beral[1], S. Evans[1], H. Shaw[2] & G. Milton[2]

[1]*Department of Epidemiology, London School of Hygiene and Tropical Medicine, London WC1E 7HT, UK;*
[2]*Department of Surgery, The University of Sydney, Sydney 2007, New South Wales, Australia.*

**Summary** In a case control study of 287 women aged 15–24 years with malignant melanoma and 574 matched controls, findings relating to oral contraceptive use and other hormone use are reported. Ever having used oral contraceptives was not associated with an increased risk of melanoma (relative risk for ever use of the pill = 1.0). Women with melanoma were, however, more likely to have taken oral contraceptives for long periods of time in the past, the relative risk associated with oral contraceptive use for a total duration of 5 years or longer which had begun at least 10 years before the melanoma was diagnosed being 1.5 (95% confidence interval 1.03 to 2.14) This elevated risk persisted after controlling for the reported hair and skin colour, frequency of moles on the body, place of birth, and measures of sunlight and fluorescent light exposure. Cases were more likely than controls to have used hormones to regulate their periods, hormonal replacement therapy and be given hormone injections to suppress lactation, the respective relative risks being 1.9, 1.4 and 1.4, but none differed significantly from 1.0. These findings suggest that prolonged oral contraceptive use may, after a lag of 10 years or so, increase the risk of malignant melanoma.

A number of recent studies have reported on the relationship between past oral contraceptive use and malignant melanoma (Beral *et al.*, 1977; Ramcharan *et al.*, 1981; Adam *et al.*, 1981; Kay, 1981; Bain *et al.*, 1982; Holly *et al.*, 1983). Most reported a weak association or none at all for melanoma and ever having used oral contraceptives. In contrast, however, all four which examined data on prolonged oral contraceptive use reported an increased risk associated with long term pill use (Beral *et al.*, 1977; Adam *et al.*, 1981; Bain *et al.*, 1982; Holly *et al.*, 1983), although the increase was not always statistically significant. We report here the findings of a case control study carried out in New South Wales, Australia, designed to examine these relationships.

### Methods

The study design has been described elsewhere (Beral *et al.*, 1982). Briefly, 287 white women attending the melanoma clinic at Sydney Hospital aged 18–54 years and 574 age-matched controls were interviewed by trained interviewers using a standard questionnaire. The diagnosis of melanoma was by biopsy and histological features were classified by the late Prof. V.J. McGovern. Two hundred and thirteen cases were "old cases" whose melanoma was diagnosed between 1974 and June 1978, when our study began. Another 87 women eligible for inclusion as old cases were not interviewed: 40 had died and 3 were too ill to be interviewed; 16 lived in very remote areas; 16 could not be traced; 4 refused to take part; and another 8 were not interviewed for a variety of reasons. The other 74 "new cases" were interviewed between June 1978 and December 1980. Two age-matched controls were chosen for each case. Controls for "old cases" were matched by area of residence and chosen from the general population; controls for "new cases" were selected from hospital inpatients, but excluded women admitted with any vascular disease or gynaecological disorder, diabetes, gallbladder or breast disease, rheumatoid arthritis, mental illness, or any chronic disease of more than 2 years' duration. The questionnaire included questions about demographic and social factors. Each woman was also asked detailed questions about her pregnancy history, and about use of oral contraceptives and of other female sex hormones. For each case the number of pregnancies she reported and the total months that she had used oral contraceptives up to the date of diagnosis of her melanoma was calculated; for the controls, number of pregnancies and oral contraceptive use was calculated up to a corresponding date. Data were analysed using the computing facilities at London University, and relative risks (RRs), chi-squared tests for trend and Mantel-Haenszel adjusted RRs estimated using the calculator programs of Rothman & Boice (1979).

### Results

The age distribution of cases was similar. The respective percentages aged 18–24 years were 14.3% and 14.3%; 24–35 years 32.1% and 33.6%; 35–44 years 35.2% and 34.8%; and 45–54 years 18.5% and 17.2%. A similar proportion (28%) of cases and controls had never used oral contraceptives

Correspondence: V. Beral
Received 4 July 1984; accepted 22 August 1984.

© The Macmillan Press Ltd., 1984

(Table I). A greater proportion of women with melanoma had taken oral contraceptives for more than 5 years (29.3% *versus* 24.3%), but this difference was not significantly different. Among those who had taken the pill, a greater percentage of cases than controls had begun taking the pill at least 10 years earlier (46.6% compared with 38.5%, $\chi^2=3.8$, $P=0.05$) Since total duration of oral contraceptive use is closely correlated with the time since first taking the pill, we examined the data simultaneously for these two factors. The only group in which there was a consistently increased risk of melanoma was in those who had begun taking the pill at least 10 years before, and who had taken it for a total duration of 5 years or longer (RR = 1.5, 95% confidence interval 1.03 to 2.14). Roughly similar relationships were found for "new" cases and "old" cases, as is shown in Table II, and so most analyses pool the two groups. Our other analyses of these data have related melanoma to a number of factors (Beral *et al.*, 1982, 1983). We therefore stratified the findings for oral contraceptives by a large number of possible confounding factors. After adjusting for marital status, hair colour, skin colour, eye colour, country of birth, number of moles on the body, educational status, exposure to fluorescent light, history of cholasma, extent of outdoor activity at ages 10, 20, 30 and 40 years, and history of sunburning various parts of the body, the relative risks associated with pill use for 5 years or more which began at least 10 years ago fluctuated between 1.43 and 1.58 and remained statistically significantly elevated. There was no significant difference in the site of the lesion, the tumour thickness nor the tumour type in women who had used the pill and those who had not (Table III).

Data on the use of other female sex hormones are shown in Table IV. The numbers reported to have ever used any of these is small, but the proportions of cases who reported that they used each was higher than the corresponding figures for the controls. The RRs and 95% confidence intervals for use of hormones to regulate periods was 1.9 (0.85–4.12). for hormones replacement therapy 1.4 (0.78–2.61) and for hormones to suppress lactation 1.3 (0.92–1.82).

Data on oral contraceptive use had been recorded for old cases on the melanoma clinic records before this survey began. The findings from our special survey agreed well with the distribution of duration of contraceptive use recorded in the clinic notes (Table V). Furthermore, data from the clinic records on oral contraceptive use in the 87 women who were eligible but not interviewed in this survey, showed that their contraceptive use was also similar to that of the other women (Table V).

**Table I** Reported oral contraceptive use in cases and controls

| *Total duration of oral contraceptive use* | *Cases* no. (%) | *Controls* no. (%) |
|---|---|---|
| Never | 79(27.5) | 159(27.8) |
| 1–11 months | 29(10.1) | 73(12.8) |
| 1–4 years | 95(33.1) | 201(35.1) |
| 5–9 years | 56(19.5) | 103(18.0) |
| 10+ years | 28 (9.8) | 36 (6.3) |
| Total | 287(100) | 572(100)[a] |

[a]Data missing for 2 controls.

**Table II** Reported duration of oral contraceptive use in "new" and "old" cases and in their corresponding controls

| *Oral contraceptive use* | *"New" cases* no. (%) | *Controls* no. (%) | *"Old" cases* no. (%) | *Controls* no. (%) |
|---|---|---|---|---|
| Use for a total duration of 5 years or longer, beginning at least 10 years before diagnosis | 18(24) | 21(14) | 41(19) | 64(15) |
| Other use | 40(54) | 80(54) | 109(51) | 248(58) |
| Never use | 16(22) | 47(32) | 63(30) | 112(26) |
| | 74(100) | 148(100) | 213(100) | 424(100) |

### Discussion

The findings presented here indicate after a lag of 10 years oral contraceptive use for a total of 5 years or longer was associated with a 50% increase in risk (RR = 1.5, 95% confidence limits 1.03–2.14). This relationship persisted after adjusting for a number of potential confounding factors, including complexion, sunbathing activities, occupation and education.

Our data on oral contraceptive use are unlikely to be biased. For old cases they agree closely with that which was recorded in the melanoma clinic records before this survey began. Furthermore, the distribution of oral contraceptive use in the 87 women who had died, could not be traced, or were not interviewed for a number of other reasons was similar to that of those who were interviewed. Only limited information about brand of oral contraceptive was collected, and no marked associations were noted.

These Australian findings on the relationship between oral contraceptives and other hormones are in general consistent with reports from the UK and USA. All studies which have examined for an association with long term pill use or for a possible

**Table III** Site of melanoma, tumour thickness and histogenetic type, according to reported oral contraceptive use. (Data on site of lesion missing for 9 cases, on thickness missing for 105, and on tumour type missing for 45 cases.)

| *Tumour characteristics* | *Oral contraceptive use* | | | |
|---|---|---|---|---|
| | *Total use of 5 years or more beginning at least 10 years before diagnosis* | *Other use* | *Never use* | *Total (=100%)* |
| *Site* | | | | |
| Head and neck | 5(23) | 10(45) | 7(32) | 22 |
| Arms | 13(25) | 26(50) | 13(25) | 52 |
| Legs | 28(19) | 70(48) | 48(32) | 146 |
| Trunk | 11(19) | 40(69) | 7(12) | 58 |
| *Thickness* | | | | |
| <0.5 mm | 7(19) | 23(62) | 7(19) | 37 |
| 0.51–1.00 mm | 17(23) | 40(55) | 16(22) | 73 |
| 1.01–2.00 mm | 7(13) | 31(57) | 16(30) | 54 |
| >2.00 mm | 4(22) | 7(39) | 7(39) | 18 |
| *Type* | | | | |
| Superficial spreading | 41(21) | 107(55) | 48(24) | 196 |
| Nodular | 8(19) | 25(60) | 9(21) | 42 |
| Hutchinson's melanotic freckle | 0(0) | 2(50) | 2(50) | 4 |
| All | 59(20.5) | 149(51.9) | 79(27.5) | 287(100) |

Percentages in parentheses.

**Table IV** Reported use of various types of hormones in cases and controls and estimated relative risk

| | *Cases number(%)* | *Controls number(%)* | *Relative risk* |
|---|---|---|---|
| Use of hormones to regulate periods | 12(4.1) | 13(2.3) | 1.9 |
| Use of hormone replacement therapy | 19(6.6) | 27(4.7) | 1.4 |
| Use of hormonal injections to suppress lactation (ever pregnant women only | 93(43.7) | 173(36.3) | 1.4 |

lag effect, have noted an increase in risk, although the numbers of cases in other studies were often small. The findings of the various studies are summarised in Table VI. Relative risk estimates reported by us or others for long duration of oral contraceptive use are in the range of 1.4 to 4.4. These relative risks are not particularly large, but they are of the same order of magnitude as the relative risks associated with characteristics such as blonde hair colour (RR = 1.6) or fair skin (RR = 2.1), noted by us for the Australian women (Beral *et al.*, 1983). Both these factors are widely accepted as being important determinants of melanoma.

Data on the use of female sex hormones, other than oral contraceptives, also tends to show a weak association with melanoma. In California the relative risk associated with other oestrogen use was 1.8 (Beral *et al.*, 1977); in Washington State it was approximately 1.0 (Holly *et al.*, 1983); in

**Table V** Comparison of duration of oral contraceptive use reported in our survey and that recorded in clinic notes

| | *213 old cases interviewed both in the clinic and in our survey* | | *87 women eligible for our survey but not interviewed* |
|---|---|---|---|
| *Duration of oral contraceptive use* | *Reported in our survey* | *Recorded in clinic notes* | *Recorded in clinic notes* |
| Never use | 63(30) | 68(32) | 24(29) |
| Total duration <5 y | 89(42) | 80(38) | 40(48) |
| Total duration ≥5 y | 61(29) | 62(30) | 20(24) |
| Missing | 0 | 3 | 3 |

Percentages in parentheses.

**Table VI** Summary of findings from different studies on melanoma and oral contraceptive use

| | *Relative risk* | |
|---|---|---|
| | *Ever use of oral contraceptives versus never use* | *Long term use of oral contraceptives versus shorter term use or never use*† |
| *Case control studies* | | |
| Beral *et al.* (1) | 1.9 | No data |
| Adam *et al.* (3)* | 1.1 | 1.6 |
| Adam *et al.* (3)† | 1.34 | 1.4 |
| Bain *et al.* (5) | 0.93 | 3.0* |
| Holly *et al.* (6) | 1.15 | 4.4* |
| This study | 1.0 | 1.5* |
| *Cohort studies* | | |
| Beral *et al.* (1) | 1.4 | 1.7 |
| Adam *et al.* (3) | 0.3 | No data |
| Kay *et al.* (4) | 1.5 | No data |
| Ramcharan *et al.* (2) | 3.5* | No data |

*Differs significantly from 1.0 ($P<0.05$).
†Long term use: definitions.
Beral *et al.*: total duration of use of 4+ years.
Adam *et al.* : total duration of use of 5+ years; a = data from postal survey; b = data from GP records.
Bain *et al.*: total duration of use of 2+ years beginning 10+ years before diagnosis.
Holly *et al.*: total duration of use of 5+ years beginning 12+ years before diagnosis (superficial spreading melanoma only).
This study: total duration of use of 5+ years, beginning 10+ years before diagnosis.

England, 1.4 (Adam *et al.*, 1981), and in our study the figure was 1.6. While none is statistically significant, all except for the Washington data show a consistent increase in risk.

While oral contraceptives and other exogenous sex hormones are clearly not major determinants of melanoma, the accumulating evidence suggests that they may increase the risk of disease. If, as our data and that of others suggest, a lag period of 10 years or more is involved, it may still be several decades before the effect of oral contraceptives on malignant melanoma can be properly evaluated.

We thank the Bureau of Statistics in Australia for assistance in selecting the controls; the interviewers and subjects who participated in the study; and Helen Edwards for typing the manuscript. The study was funded by the NICHD grant number 1-HD-8-2804.

### References

ADAM, S.A., SHEAVES, J.K., WRIGHT, N.H., MOSSER, G., HARRIS, R.W. & VESSEY, M.P. (1981). A case control study of the possible association between oral contraceptives and malignant melanoma. *Br. J. Cancer*, **44**, 45.

BAIN, C., HENNEKENS, C.H., SPEIZER, F.E., ROSNER, B., WILLETT, W. & BELANGER, C. (1982). Oral contraceptive use and malignant melanoma. *J. Natl Cancer Inst.*, **68**, 537.

BERAL, V., EVANS, S., SHAW, H. & MILTON, G. (1982). Malignant melanoma and exposure to fluorescent lighting at work. *Lancet*, **ii**, 290.

BERAL, V., EVANS, S., SHAW, H. & MILTON, G. (1983). Cutaneous factors related to the risk of melanoma. *Br. J. Dermatol.*, **109**, 165.

BERAL, V., RAMCHARAN, S. & FARIS, R. (1977). Malignant melanoma and oral contraceptive use among women in California. *Br. J. Cancer*, **36**, 804.

HOLLY, E.A., WEISS, N.S. & LIFF, J.M. (1983). Cutaneous melanoma in relation to exogenous hormones and reproductive factors. *J. Natl Cancer Inst.*, **70**, 827.

KAY, C.R. (1981). Malignant melanoma and oral contraceptives. *Br. J. Cancer*, **44**, 479.

RAMCHARAN, S., PELLEGRIN, F.A., RAY, R. & HSU, J.P. (1981). *The Walnut Creek Contraceptive Drug Study. A prospective study of the side effects of oral contraceptives*. Volume III. US Government Printing Office, Washington DC.

ROTHMAN, K.J. & BOICE, J.D. (1979). *Epidemiologic Analysis with a Programmable Calculator*. Washington DC: Government Printing Office, (DHEW publication no. 1 (NIH) 79-1649).